# Effects of Cannabidiol and Hypothermia on Short-Term Brain Damage in New-Born Piglets after Acute Hypoxia-Ischemia

**DOI:** 10.3389/fnins.2016.00323

**Published:** 2016-07-12

**Authors:** Hector Lafuente, Maria R. Pazos, Antonia Alvarez, Nagat Mohammed, Martín Santos, Maialen Arizti, Francisco J. Alvarez, Jose A. Martinez-Orgado

**Affiliations:** ^1^Neonatology Research Group, Biocruces Health Research InstituteBizkaia, Spain; ^2^Group of Cannabinoids Research on Neonatal Pathologies, Research Institute Puerta de Hierro MajadahondaMadrid, Spain; ^3^Department of Cell Biology, University of the Basque CountryLeioa, Spain; ^4^Department of Neonatology, Hospital Clínico San Carlos–Instituto de Investigación Sanitaria San Carlos (IdISSC)Madrid, Spain

**Keywords:** hypothermia, neonatal brain, neurodevelopment, neuroprotection, hypoxic-ischemic encephalopathy, newborn animal, cannabidiol

## Abstract

Hypothermia is a standard treatment for neonatal encephalopathy, but nearly 50% of treated infants have adverse outcomes. Pharmacological therapies can act through complementary mechanisms with hypothermia improving neuroprotection. Cannabidiol could be a good candidate. Our aim was to test whether immediate treatment with cannabidiol and hypothermia act through complementary brain pathways in hypoxic-ischemic newborn piglets. Hypoxic-ischemic animals were randomly divided into four groups receiving 30 min after the insult: (1) normothermia and vehicle administration; (2) normothermia and cannabidiol administration; (3) hypothermia and vehicle administration; and (4) hypothermia and cannabidiol administration. Six hours after treatment, brains were processed to quantify the number of damaged neurons by Nissl staining. Proton nuclear magnetic resonance spectra were obtained and analyzed for lactate, N-acetyl-aspartate and glutamate. Metabolite ratios were calculated to assess neuronal damage (lactate/N-acetyl-aspartate) and excitotoxicity (glutamate/Nacetyl-aspartate). Western blot studies were performed to quantify protein nitrosylation (oxidative stress), content of caspase-3 (apoptosis) and TNFα (inflammation). Individually, the hypothermia and the cannabidiol treatments reduced the glutamate/Nacetyl-aspartate ratio, as well as TNFα and oxidized protein levels in newborn piglets subjected to hypoxic-ischemic insult. Also, both therapies reduced the number of necrotic neurons and prevented an increase in lactate/N-acetyl-aspartate ratio. The combined effect of hypothermia and cannabidiol on excitotoxicity, inflammation and oxidative stress, and on cell damage, was greater than either hypothermia or cannabidiol alone. The present study demonstrated that cannabidiol and hypothermia act complementarily and show additive effects on the main factors leading to hypoxic-ischemic brain damage if applied shortly after the insult.

## Introduction

Excitotoxicity, inflammation and oxidative stress are the “deadly triad” leading to hypoxia-ischemia (HI)-induced brain damage (Johnston et al., 2011; Drury et al., 2014; Juul and Ferriero, 2014). Only pleiotropic therapies acting on all these processes provide successful neuroprotection (Cilio and Ferriero, 2010; Johnston et al., 2011; Juul and Ferriero, 2014). Therapeutic hypothermia is an example of a pleiotropic neuroprotective therapy (Drury et al., 2014). Studies in immature an adult animals have demonstrated antiexcitotoxic, anti-oxidant, and anti-inflammatory effects of hypothermia (Thoresen et al., 1997; Mueller-Burke et al., 2008; Jenkins et al., 2012; Joseph et al., 2012; Sandu et al., 2016) which become the gold standard for treating infants with hypoxic-ischemic encephalopathy (HIE; Jacobs et al., 2013). Despite treatment with hypothermia, some infants with neonatal HIE present adverse outcomes. Further trials to determine the appropriate techniques of cooling, including refinement of patient selection, duration of cooling and method of providing therapeutic hypothermia, will improve our understanding of this therapy (Jacobs et al., 2013). In addition, a recent meta-analysis reported that hypothermia is not protective and can even be harmful in low- and middle-income countries in Africa and Asia (Pauliah et al., 2013). In this context, complementary therapies can be combined with hypothermia to enhance its neuroprotective properties and/or extend its therapeutic time window. These therapies might even become an alternative to hypothermia when needed (Cilio and Ferriero, 2010). On the other hand, a successful treatment for HIE neuroprotection may not be complementary to hypothermia if its mechanisms of action overlap with those of hypothermia without any improvement of the outcome. Thus, IGF-1 (George et al., 2011) or SOD mimetic EUK-134 (Ni et al., 2012) therapies failed to demonstrate additive neuroprotective effects when combined with hypothermia. By contrast, other therapies such as xenon or melatonin (Chakkarapani et al., 2010; Faulkner et al., 2011; Robertson et al., 2013), have been observed to augment hypothermic neuroprotection at least in some situations, showing additive protection with mild hypothermia.

Cannabidiol (CBD), the main non-psychoactive component of Cannabis sativa, has recently been included among pleiotropic therapies, as it has been shown to reduce HI-induced brain damage in newborn rats and piglets by modulating excitotoxicity, inflammation and oxidative stress (Alvarez et al., 2008; Castillo et al., 2010; Lafuente et al., 2011; Pazos et al., 2012, 2013). Some of the properties of CBD underlying these effects include its antioxidant properties, the inhibition of calcium transport across membranes, inhibition of endocannabinoid uptake and enzymatic hydrolysis, activation of serotonin 5HT1A receptors and inhibition of NF-κB activation (Alvarez et al., 2008; Castillo et al., 2010; Lafuente et al., 2011; Pazos et al., 2012, 2013). The neuroprotective action of CBD was not associated with significant side effects, and additional benefits were demonstrated if immediate rescue treatment was provided (Alvarez et al., 2008; Lafuente et al., 2011; Pazos et al., 2012, 2013). In summary, these studies support the role of CBD as a non-artificial drug for newborns with HIE. However, no evidences of how CBD might work together with hypothermia have been provided so far.

Our aim was to assess the early effects of combining cannabidiol with therapeutic hypothermia in neonatal hypoxic-ischemic piglets using clinical biomarkers of brain injury shortly after the injury.

## Materials and methods

All experimental procedures and euthanasia of the animals were conducted in strict compliance with European and Spanish regulations on the protection of animals used for scientific purposes (European Directive 2010/63/EU and Spanish Royal Legislative Decree 53/2013). The protocols were approved by the Committees on the Ethics of Laboratory Animal Welfare of Biocruces Health Research Institute and the Research Institute Puerta de Hierro Majadahonda (Permit Numbers: SEP#009_09 and SEP#012_11) and performed in its experimental surgical theaters. Using G^*^Power 3.0 (Faul et al., 2007), the change in the area under the curve for lactate/N-acetyl aspartate from baseline at 6 h was used as the primary outcome to calculate the sample size. Our previous work in this model suggested that the change in lactate/N-acetyl aspartate ratio after 6 h varied between vehicle-treated and cannabidiol-treated groups by 50 ± 20%. Assuming similar magnitude of additional effect for cannabidiol-augmented hypothermia (vs. hypothermia or cannabidiol alone) and with an alpha risk of 0.05 and a beta risk of 0.2, eight subjects would be necessary in each group. It was anticipated a drop-out rate of 5%. All experimental procedures were designed and carried out by personnel qualified in Laboratory Animal Science, following FELASA recommendations (Category B and C). All surgery was performed under adequate anesthesia and analgesia, and great efforts were made to minimize suffering.

### Experimental protocol

Animals were daily obtained from a local vendor with specific pathogen free status for international swine breeding program (TOPIG, B-line) and for *in vitro* genetic reproduction (Arri-Turri farm, Alava, Spain). In short, 1- to 2-day-old male piglets were intubated under 5% sevoflurane anesthesia and managed by controlled mechanical ventilation (VIP Bird, Bird Corp., Palm Springs, CA, USA). A marginal ear vein was cannulated to maintain intravascular anesthesia and analgesia by continuous infusion of 3 mg/kg/h propofol, 0.5 mg/kg/h midazolam, and 4 μg/kg/h fentanyl. Once adequate analgesia was achieved and confirmed, respiratory paralysis was induced with 3 mg/kg/h atracurium to prevent spontaneous breathing. Then, the two common carotid arteries were exposed and elastic bands were placed loosely around each one. A non-invasive ultrasonic probe (Transonic Systems Inc., NY) was placed in the right common carotid artery to measure the instantaneous blood flow. Indwelling catheters (5 Fr, PiCCO Plus, Pulsion Medical Systems, München, Germany) were inserted into the right jugular vein, to infuse dextrose (at a rate of 4 mg/kg/min), and into the right femoral artery to continuously monitor cardiac output, heart rate, mean arterial blood pressure and central temperature (Omnicare CMS 24, HP, Göblingen, Germany). Further, brain activity was monitored by amplitude-integrated electroencephalography (aEEG; BRM2; BrainZ Instruments, Auckland, New Zealand). The “raw” EEG traces were manually reviewed for electrical seizures. Body temperature was maintained between 37.5 and 38.5ºC using an air-warmed blanket. Arterial blood gases were monitored throughout the experiment. Dopamine infusion (10–20 μg/kg/min) was used as needed to maintain mean arterial blood pressure over 40 mmHg (Chakkarapani et al., 2010; Faulkner et al., 2011; Robertson et al., 2013). After surgical instrumentation, each animal was left to stabilize for 30 min (baseline).

After the surgery, HI brain injury was induced in the piglets by total interruption of the carotid blood flow (tightening the elastic bands around the arteries, and confirmed by the ultrasonic probe) and reducing the fraction of inspired oxygen to less than 10%. The hypoxic-ischemic conditions were maintained for 30 min, measured from the point at which there was evidence of reduced brain activity on the aEEG (flat traces < 4 μV). After this period of injury (end of HI), carotid blood flow was restored and the inspired fraction of oxygen was returned to 21%.

After 30 min, control and HI-injured piglets were first randomized by sealed envelope to normothermia or hypothermia. In normothermic animals, rectal temperature was maintained at 38ºC (range: 37.5–38.5ºC) using the air-warmed blanket. In hypothermic animals, based on previously reported studies for combination of therapies in hypoxic-ischemic piglets (Chakkarapani et al., 2010; Faulkner et al., 2011; Robertson et al., 2013), rectal temperature was reduced within 10 min to 33–34ºC using a circulating water mattress (Recirculating Chiller 1171MD, VWR International, CA, USA).

Then, HI-injured piglets treated with normothermia or hypothermia were again randomized for drug administration by sealed envelope. CBD (a generous gift by GW Pharma Ltd, Mambridge, UK) was prepared in a 5 mg/mL formulation of ethanol/solutol/saline (2:1:17), as reported in previous studies (Alvarez et al., 2008; Lafuente et al., 2011; Pazos et al., 2012, 2013). Doses were selected following previous *in vivo* experiments by our group (Pazos et al., 2012, 2013) and HI-injured animals received i.v. 1 mg/kg (0.2 mL/kg) of CBD or an equivalent volume of vehicle (ethanol/solutol/saline), further diluted to 10 mL in saline before administration as slow bolus.

After 6 h of treatment, anesthetized piglets were euthanized by cardiac arrest with potassium chloride (~150 mg/kg), and the brains were immediately removed from the skull and sliced. Brain slices were placed into 4% paraformaldehyde, for histological analysis (left hemisphere), or frozen in isopentane and conserved at −80ºC, for spectroscopy and biochemical analysis (right hemisphere). For both types of analysis, we used parietal cortex samples from the same brain section, corresponding to 5 mm of the posterior brain region identified according to the stereotaxic atlas of the pig brain (Felix et al., 1999).

Accordingly, there were four experimental groups of HI piglets: normothermic vehicle-treated group (NV; *n* = 9), normothermic cannabidiol-treated group (NC; *n* = 11), hypothermic vehicle-treated group (HV; *n* = 8) and hypothermic cannabidiol-treated group (HC; *n* = 8). Normothermic and hypothermic sham operated groups of animals without HI are included in supplemental files as referenced data.

### Histological analysis

Neuronal necrosis was identified by Nissl staining in 4-μm thick coronal sections obtained from fixed brain hemispheres, as described previously (Alvarez et al., 2008; Lafuente et al., 2011; Pazos et al., 2013). Random areas were examined in the three central lobes of the parietal cortex, focusing on 1-mm^2^ areas in the layers II-III. This analysis was conducted by a pathologist investigator blinded to the experimental groups using an optical microscope at 400x and a grid of 50 compartments, with the mean of three compartments being calculated. The parietal cortex was selected to illustrate histological brain damage because this is the most vulnerable area in the brain to HI in piglets, and because the damage seen in this area correlates with that observed in other vulnerable areas such as the hippocampus or striatum (Peeters-Scholte et al., 2003; Schubert et al., 2005). Normal neuronal cells were recognized by the presence of the typical nuclei with clear nucleoplasm and distinct nucleolus surrounded by purple-stained cytoplasm containing Nissl substance. Neurons were classified as damaged cells (pyknotic or necrotic) when we were unable to ascertain a well-defined nuclear and cytoplasmic boundary.

### Proton magnetic resonance spectroscopy (H^+^-MRS)

*Ex vivo* proton magnetic resonance spectroscopy (H^+^-MRS) has been demonstrated to provide metabolic information with higher sensitivity and spectral resolution than *in vivo* magnetic resonance spectroscopy (Jiménez-Xarrié et al., 2015). As previously described (Pazos et al., 2013), H^+^-MRS was performed in the MRI Unit of the Instituto Pluridisciplinar (Universidad Complutense, Madrid, Spain) at 500.13 MHz using a Bruker AMX500 spectrometer at 11.7 T, operating at 4ºC on frozen parietal cortical samples (5–10 mg weight) placed within a 50-μL zirconium oxide rotor with a cylindrical insert and spun at a rate of 4000 Hz. H^+^-MRS spectra were acquired using a standard solvent-suppressed pulse/acquire sequence based on the start of a Nuclear Overhauser Effect Spectroscopy pulse sequence, with 16,000 data points, averaged over 256 acquisitions. Data acquisition time was approximately 14 min. The NOESY preset pulse sequence employed a mixing time of 150 ms with t1 fixed at 3 μs, during which time the effects of B0 and B1 field inhomogeneities were suppressed (relaxation delay-90º-t1-90º-tm-90º-acquire free induction decay signal). A secondary radio-frequency field was applied at the water resonance frequency during the relaxation delay of 2 s.

A spectral width of 8333.33 Hz was used. All spectra were processed using TOPSPIN software, version 1.3 (Bruker Rheinstetten, Germany). Prior to Fourier transformation, the free induction decay values were multiplied by an exponential weight function corresponding to a line broadening of 0.3 Hz. Spectra were phased, baseline-corrected and referenced to the sodium (3-trimethylsilyl)-2,2,3,3-tetradeuteriopropionate singlet at δ = 0 ppm.

After curve fitting was performed (SpinWorks ver. 3.1.7.0, University of Manitoba, Canada), all spectra were analyzed for lactate (Lac), N-acetyl-aspartate (NAA), and glutamate (Glu). Later, several ratios were calculated that are predictive of neurodevelopmental outcome after hypoxic-ischemic brain damage in piglets (Vial et al., 2004; Munkeby et al., 2008): lactate/N-acetylaspartate (Lac/NAA) and glutamate/N-acetylaspartate (Glu/NAA).

### Western blot studies

Levels of oxidized proteins were quantified by Western blot analysis to assess protein carbonylation in brain tissue. A detection kit (Millipore Iberica; Madrid, Spain) was used according to the manufacturer's protocol, and brain samples containing 15 μg of total protein were subjected to the derivatization reaction with 2,4-dinitrophenylhydrazine (DNPH). Two aliquots of each sample to be analyzed were treated: one aliquot was subjected to derivatization reaction, and the other aliquot, served as a negative control, by substituting the derivatization control solution with DNPH solution. The treated samples were fractionated in a 12% dodecyl-sulfate–polyacrylamide gel and electroblotted onto PVDF membranes (GE Healthcare; Buckinghamshire, UK). DNPH-BSA standards (Millipore Iberica, Madrid, Spain) were loaded on each gel and used as a reference. Membranes were incubated in Tris/glycine/methanol transfer buffer at 4ºC and proteins separated under constant voltage (2 h at 250 mA). The resulting blots were blocked by overnight incubation in phosphate-buffered saline-Tween (PBST) containing 5% nonfat dried milk at 4ºC. Primary antibody incubation (Rabbit Anti-DNPH 1:150; Millipore Iberica, Madrid, Spain) was performed in PBST containing 5% nonfat dried milk for 1 h at room temperature. After washing with PBST, the membranes were incubated with the secondary antibody (Goat anti-rabbit IgG HRP conjugated 1:300; Millipore Iberica, Madrid, Spain) for 1 h at room temperature. Finally, an enhanced chemiluminescent substrate detection system (GE Healthcare, Buckinghamshire, UK) was used to visualize the blots. The films were scanned and analyzed with ImageJ software (NIH, USA). Oxidized protein levels were quantified via measurement of the optical density, using the NIH Image J analysis software (Bethesda, MD, USA). Results were normalized by total protein loading (Red Ponceau staining) and expressed as OxyBlot/Red Ponceau ratio.

TNFα and caspase-3 Western blot assays were performed with brain samples containing 20 μg of total protein. In this case, proteins were electrophoresed in an 18% sodium dodecyl-sulfate–polyacrylamide gel and then electroblotted onto PVDF membranes (GE Healthcare, Buckinghamshire, UK) in Tris/glycine/methanol transfer buffer under constant voltage (2 h at 250 mA). The resulting blots were blocked in PBST containing 5% nonfat dried milk at room temperature for 1 h, followed by primary antibody incubation (Rabbit anti-TNFα 1:1000, and rabbit anti-Caspase-3 1:500; Abcam Plc., Cambridge, UK) in PBST containing 5% nonfat dried milk at 4ºC overnight. After washing with PBST, the membranes were incubated with the secondary antibody (Goat anti-rabbit IgG HRP-Conjugated for TNFα and goat anti-mouse IgG HRP-Conjugated for caspase-3, 1:2000; Bio-Rad Lab., Hercules, CA, USA) for 1 h at room temperature. Finally, as above, the ECL system was used, and the films were scanned and analyzed using ImageJ software. Protein levels were quantified using densitometric analysis, normalized by β-actin (1:500, Abcam Plc., Cambridge, UK) loading and expressed as TNFα/β-actin and caspase-3/β-actin ratios.

### Statistical analyses

SPSS 19.0.0 software (IBM Software, NY, USA) was used for statistical analyses. All data are expressed as mean ± standard error of the mean (SEM). Comparisons between groups were performed by Kruskall-Wallis one-factorial analysis of variance by ranks with Dunn's *post-hoc* test. A *P* < 0.05 was considered to be statistically significant.

## Results

**Figure 1:**
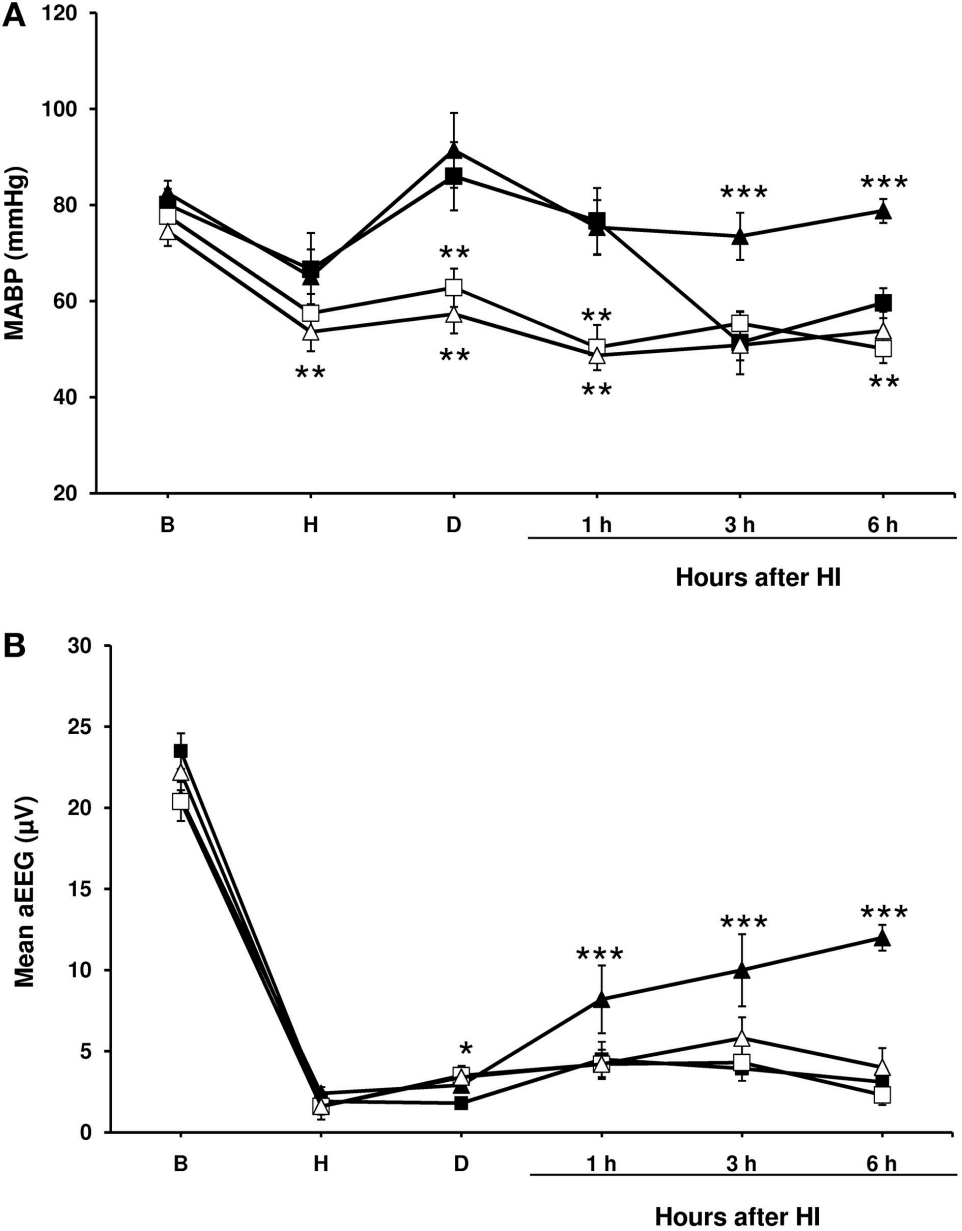
**Mean arterial blood pressure and amplitude-integrated electroencephalography (aEEG) in experimental groups of piglets**. Line draws represent the mean arterial blood pressure **(A)** and the mean aEEG **(B)**, obtained from 1- to 2-day-old piglets after hypoxic-ischemic insult, treated with normothermia (shaded symbol) or hypothermia (empty symbol) and administered with either vehicle (square) or cannabidiol (triangle). Data are represented as mean ± SEM. (^*^) *P* < 0.05 vs. NV group by Kruskall-Wallis one-factorial analysis of variance; (^**^) *P* < 0.05 vs. both normothermic groups (NV and NC) by Kruskall-Wallis one-factorial analysis of variance; (^***^) *P* < 0.05 vs. all groups by Kruskall-Wallis one-factorial analysis of variance.

Normothermic and hypothermic sham operated groups of animals without HI are included in the Supplementary Materials. Piglets from all experimental groups were similar in terms of age, weight and physiological parameters (Table 1), except for mean arterial blood pressure and aEEG (Figure 1). The hypoxic-ischemic insult induced a decrease in pH in all injured animals (H interval, Table 1). After hypothermic therapy begins, pH levels significantly recovered toward baseline levels at the drug treatment (D interval, Table 1) in both HV and HC groups. At the end of experiment (E interval, Table 1), mean pH level in HC group was significantly higher than in the other groups. In normothermia-treated animals, the hypoxic-ischemic insult led to a decrease in mean arterial blood pressure that spontaneously recovered in the following 30 min.

**Table 1:** **General and physiological parameters**.

**Group**		**NV**	**NC**	**HV**	**HC**
**Sample size**		**(*n* = 9)**	**(*n* = 11)**	**(*n* = 8)**	**(*n* = 8)**
Age (days)		1.8 (0.1)	1.9 (0.1)	1.6 (0.1)	1.7 (0.1)
Weight (kg)		1.7 (0.1)	1.9 (0.1)	1.6 (0.1)	1.6 (0.1)
CO (ml/min/100 g)	B	339 (35)	357 (25)	315 (11)	301 (41)
	H	355 (34)	321 (66)	410 (28)	402 (32)
	D	369 (41)	334 (41)	317 (15)	262 (26)
	E	344 (42)	336 (43)	324 (27)	292 (20)
pH	B	7.32 (0.02)	7.35 (0.02)	7.36 (0.01)	7.41 (0.03)
	H	7.17 (0.04)	7.20 (0.04)	7.23 (0.04)	7.24 (0.03)
	D	7.21 (0.03)	7.20 (0.05)	7.36 (0.03)^**^	7.40 (0.03)^**^
	E	7.32 (0.03)	7.32 (0.02)	7.34 (0.02)	7.42 (0.03)^***^
pCO_2_ (mm Hg)	B	39.4 (1.9)	40.7 (2.6)	40.5 (1.5)	39.4 (2.6)
	H	39.2 (2.9)	40.1 (3.3)	43.4 (1.5)	44.4 (2.9)
	D	42.4 (2.2)	43.4 (2.3)	37.4 (3.2)	38.0 (3.0)
	E	42.2 (2.9)	42.3 (1.6)	43.3 (1.8)	37.2 (2.2)

Mean values ± SEM (given in brackets) obtained from 1- to 2-day-old piglets after hypoxic-ischemic insult, treated with normothermia or hypothermia and administered with either vehicle or cannabidiol. B, Baseline; H, end of HI, D, Drug; E, End of the experiment (^**^) P < 0.05 vs. both normothermic groups (NV and NC) by Kruskall-Wallis one-factorial analysis of variance; (^***^) P < 0.05 vs. all groups by Kruskall-Wallis one-factorial analysis of variance.

In the NV group mean arterial blood pressure values dropped again throughout the post-HI period (Figure 1A); in a half of the animals (5 animals), dopamine infusion (at 10–20 μg/kg/min) was needed to maintain mean arterial blood pressure over 40 mmHg. Hypothermia was associated with a decrease in mean arterial blood pressure in all groups (Figure 1A), although dopamine infusion was not administered since the mean arterial blood pressure did not fall under 40 mmHg in any of the piglets. No such decline in the mean arterial blood pressure was observed in the HI-injured piglets treated with normothermia and CBD, and these animals did not require dopamine.

After injury, basal aEEG amplitude was decreased below 4 μV in all normothermic and hypothermic groups (Figure 1B). The depressed activity remained unchanged in both NV, HV and HC groups during all experiment, but was partially recovered at the end of the experiment in NC group. Electrical seizures were detected in one piglet from the HV group and one from the HC group.

### Effects of the treatments on the characteristics of excitotoxicity

**Figure 2:**
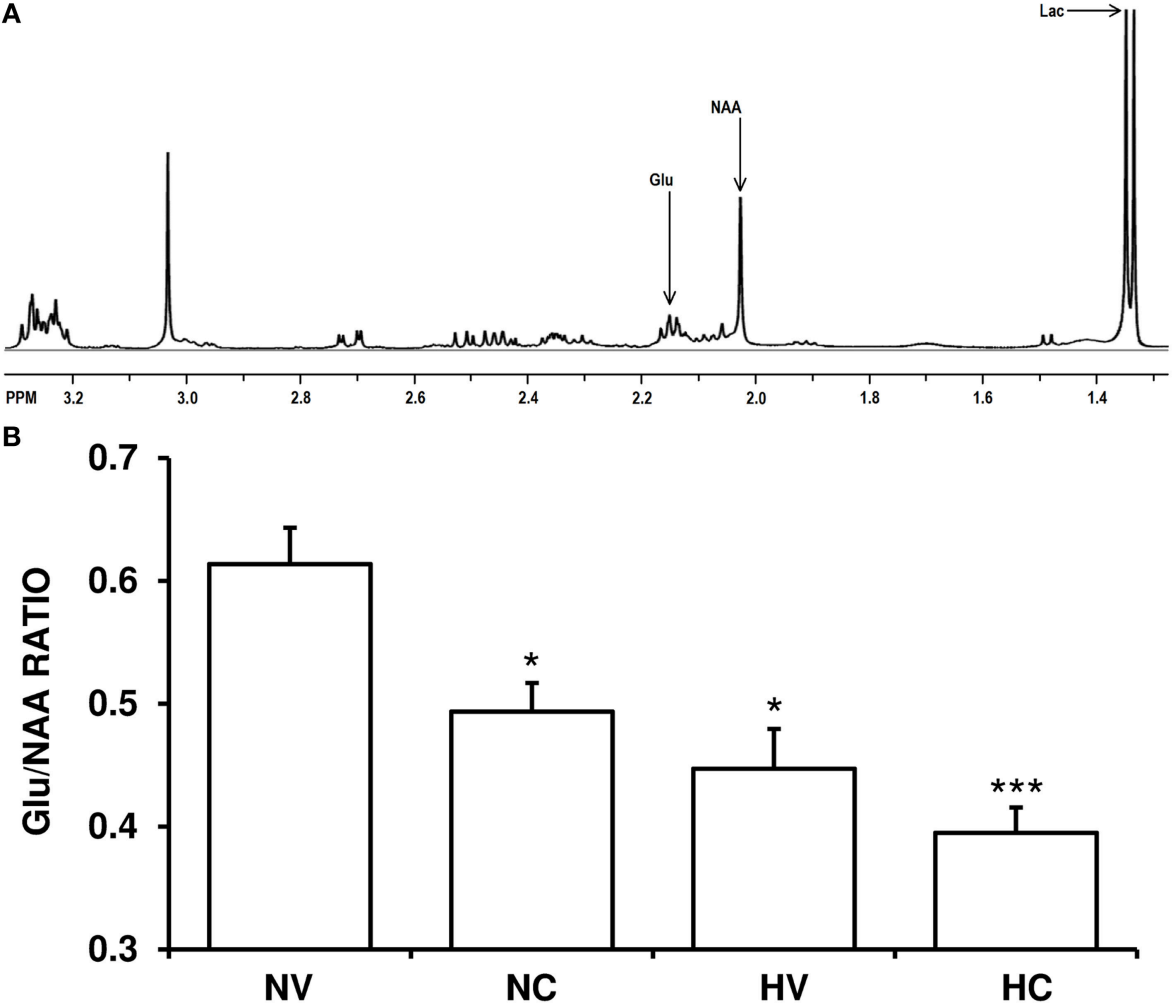
**Effect of the treatments on the characteristics of excitotoxicity. (A)** A representative brain spectra from a hypoxic-ischemic animal managed with normothermia (NV group). Arrows show the metabolite peaks of glutamate (Glu), N-acetylaspartate (NAA) and lactate (Lac). **(B)** Bars represent the results of Glu/NAA ratio (mean ± SEM), in brain samples from 1- to 2-day-old piglets after hypoxic-ischemic insult, treated with normothermia or hypothermia and administered with either vehicle or cannabidiol. (^*^) *P* < 0.05 vs. NV group by Kruskall-Wallis one-factorial analysis of variance; (^***^) *P* < 0.05 vs. all groups by Kruskall-Wallis one-factorial analysis of variance.

The Glu/NAA ratio was higher in the NV group than in the HV group (Figure 2). Cannabidiol treatment alone (NC group) reduced Glu/NAA ratio to a similar extent compared to the levels in HV group. The administration of both therapies (hypothermia and CBD) together produced additive effects, so the Glu/NAA ratio in the HC group was lower to that observed in HV or NC groups.

### Effect of the treatments on the characteristics of oxidative stress

**Figure 3:**
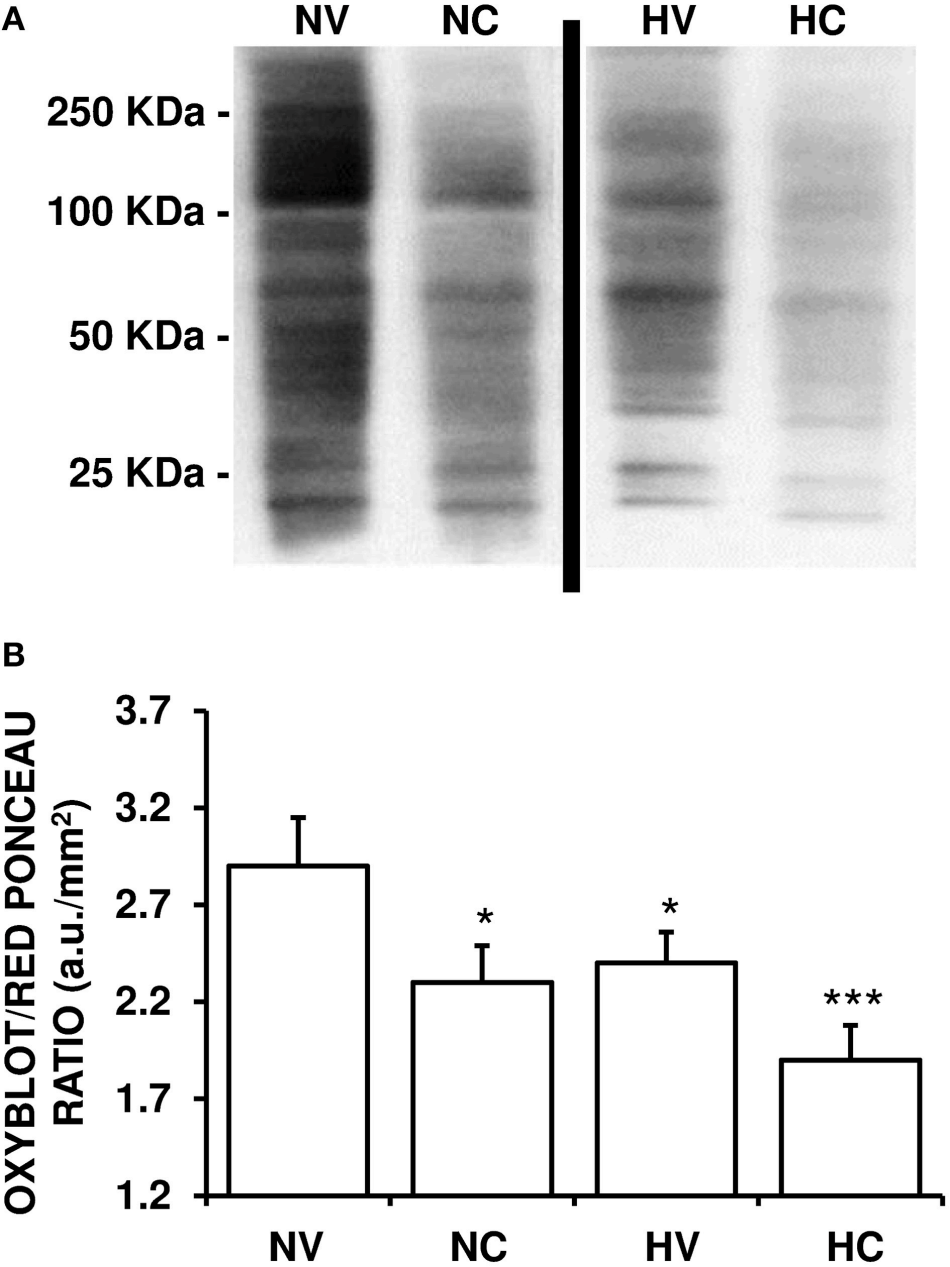
**Effect of the treatments on the characteristics of oxidative stress. (A)** A representative image of immunoblotting indicating protein carbonyl formation (Oxyblot) using anti-DNPH antibody. Samples were obtained using brain tissue from 1- to 2-day-old piglets after hypoxic-ischemic insult, treated with normothermia or hypothermia and administered with either vehicle or cannabidiol. **(B)** Relative protein carbonyl levels were quantified by densitometric analyses of the blots. The content of oxidized proteins was normalized by total Red Ponceau-stained protein loading and expressed as OxyBlot/Red Ponceau ratio (see Materials and Methods for details). Bars represent mean ± SEM of 6–8 experiments. (a.u.) arbitrary units; (^*^) *P* < 0.05 vs. NV group by Kruskall-Wallis one-factorial analysis of variance; (^***^) *P* < 0.05 vs. all groups by Kruskall-Wallis one-factorial analysis of variance.

A lower mean value of OxyBlot/Red Ponceau ratio in the analyzed proteins from the parietal cortex was observed in the HV group than in NV group (Figure 3), indicating a reduction of protein carbonylation. A single dose of CBD after HI (NC group) induced a similar effect on this ratio as in the HV group (Figure 3). The mean levels of OxyBlot/Red Ponceau ratio were lower in the HC group than in the HV or NC groups, suggesting that administration of hypothermia together with CBD led to an additive effect.

### Effect of the treatments on the characteristics of neuroinflammation

**Figure 4:**
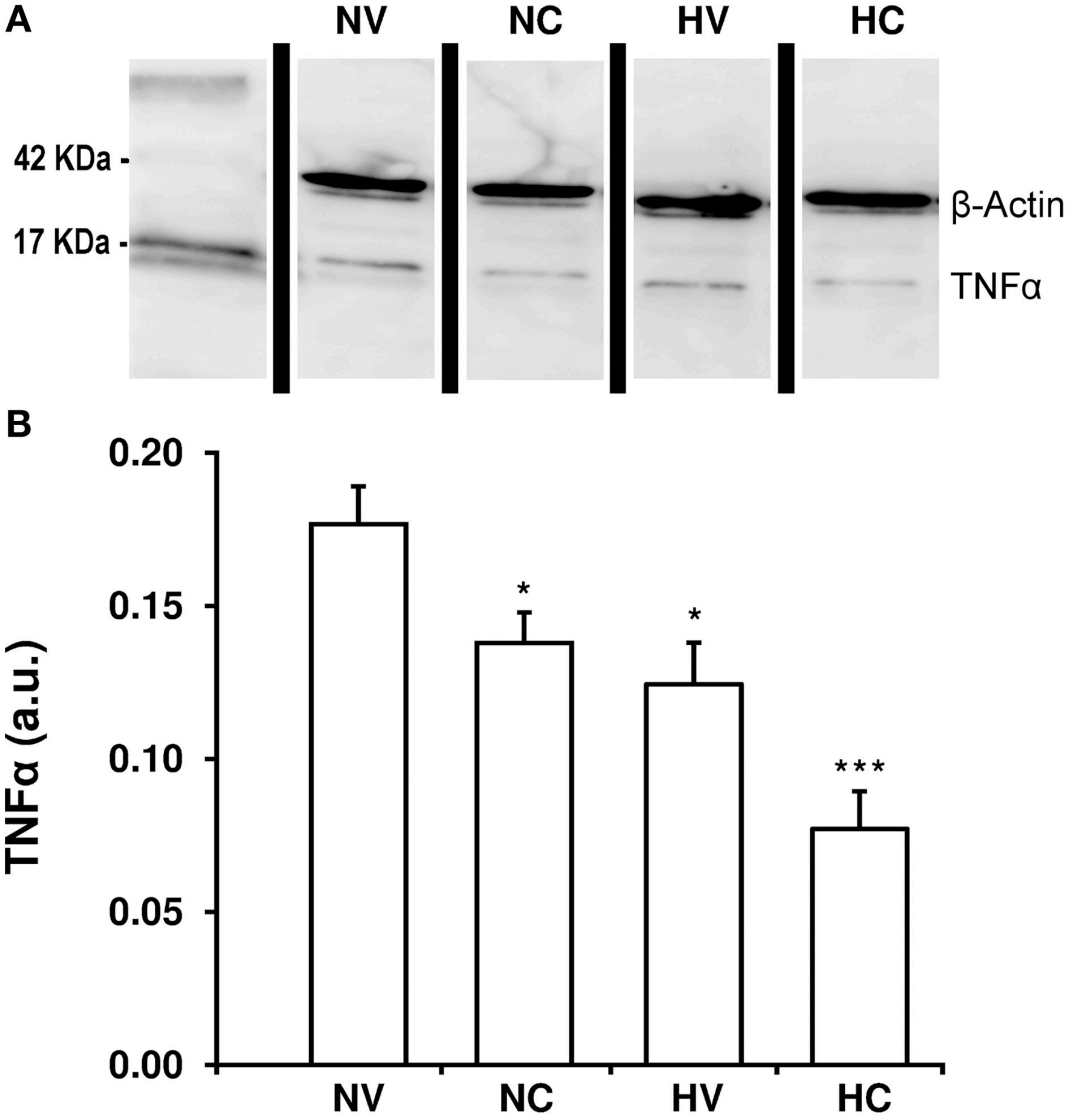
**Effect of the treatments on the characteristics of neuroinflammation. (A)** A representative image of immunoblotting using anti-TNFα antibody, carried out on the samples from 1- to 2-day-old piglets after hypoxic-ischemic insult, treated with normothermia or hypothermia and administered with either vehicle or cannabidiol. **(B)** Densitometric analysis of relative TNFα contents. β-actin was used to normalize for the differences in protein loading between lanes in the blot. Bars represent mean ± SEM of 6-8 experiments. (a.u.) arbitrary units; (^*^) *P* < 0.05 vs. NV group by Kruskall-Wallis one-factorial analysis of variance; (^***^) *P* < 0.05 vs. all groups by Kruskall-Wallis one-factorial analysis of variance.

Hypothermia treatment led to a reduction of TNFα content in the parietal cortex of animals from the HV group as compared to the NV group (Figure 4). With the administration of CBD in normothermia, brain TNFα concentration was almost 18% lower than in the NV group. Again, the combination of two treatments resulted in an additive effect: TNFα levels in the HC group were lower than those in the NC or HV groups.

### Effects of the treatments on the characteristics of brain damage

**Figure 5:**
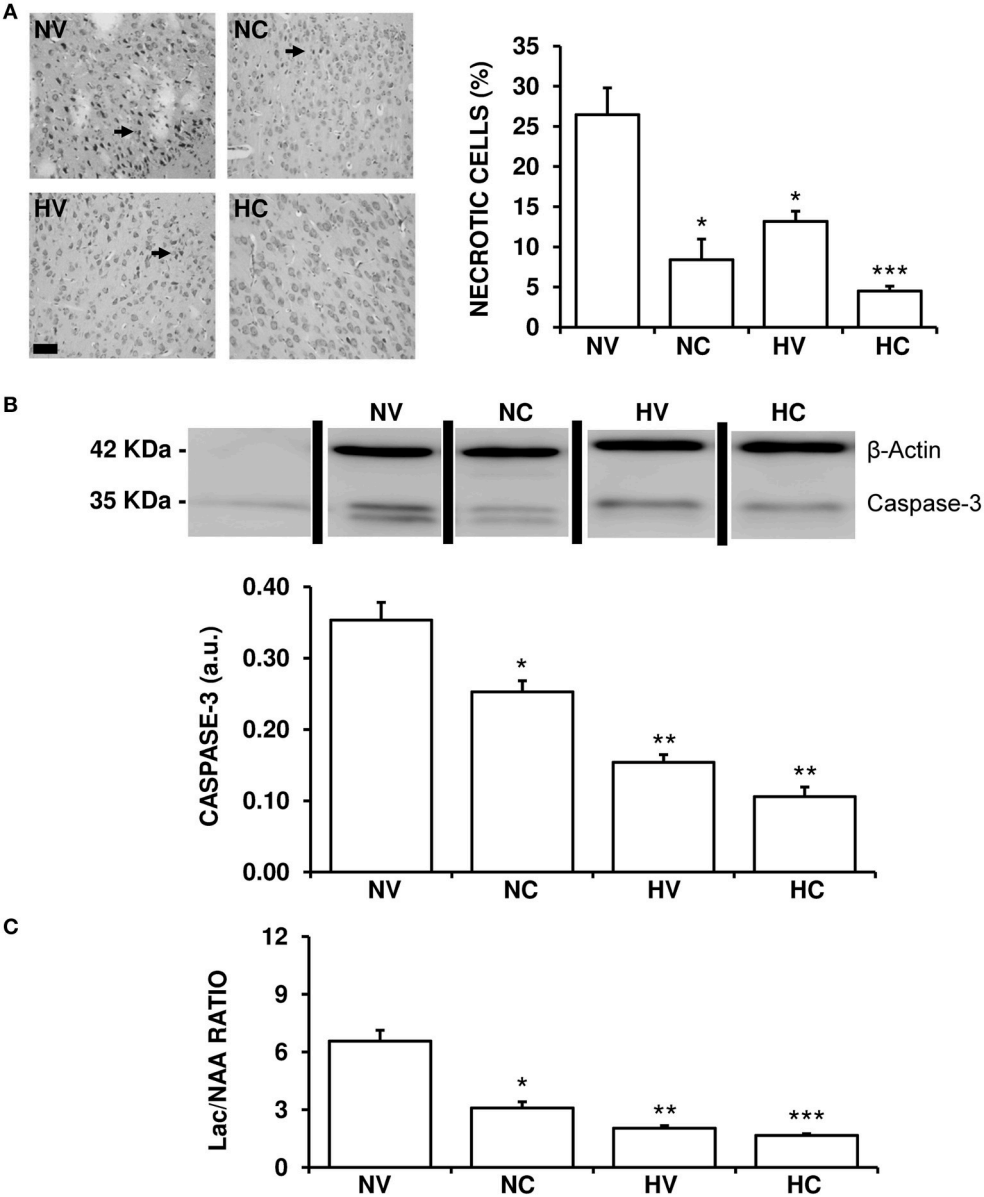
**Effect of the treatments on the characteristics of brain lesion. (A)** Representative light microphotographs of Nissl-stained brain sections, obtained from 1- to 2-day-old piglets after hypoxic-ischemic insult, treated with normothermia or hypothermia and administered with either vehicle or cannabidiol. The number of pyknotic cells was significantly higher in the NV group (arrows), while it was lower in the hypothermia and cannabidiol-treated groups. Original magnification x200, white bar: 100 μm. **(B)** Top: A representative image of immunoblotting using an anti-Caspase-3 antibody, carried out on the samples from 1- to 2-day-old piglets after hypoxic-ischemic insult, treated with normothermia, or hypothermia and administered with either vehicle or cannabidiol. Bottom: Densitometric analysis of relative caspase-3 contents. β-actin was used to normalize for differences in protein loading between lanes of the blot. **(C)** Changes in Lac/NAA ratio obtained by H^+^-MRS analysis of brain samples from 1- to 2-day-old piglets after hypoxic-ischemic insult, treated with normothermia or hypothermia and administered with either vehicle or cannabidiol. Lac: lactate; NAA: N-acetylaspartate. In all figures, bars represent mean ± SEM of 6–10 experiments. (a.u.) arbitrary units. (^*^) *P* < 0.05 vs. NV group by Kruskall-Wallis one-factorial analysis of variance; (^**^) *P* < 0.05 vs. both normothermic groups (NV and NC) by Kruskall-Wallis one-factorial analysis of variance; (^***^) *P* < 0.05 vs. all groups by Kruskall-Wallis one-factorial analysis of variance.

HI-induced brain damage and treatment-induced effects were both observed in the histological and biochemical analyses. At the end of the experiment, about a quarter of neurons in the cortex of the NV group animals appeared to be necrotic (Figure 5A). Either hypothermia treatment (HV group) or CBD administration (NC group) alone reduced this percentage to a similar extent. Administration of both therapies together produced an additive effect bringing the per cent of necrotic neurons in the HC group down compared to the NC or HV animals.

The expression of caspase-3 was reduced in the HV group in comparison to NV group (Figure 5B). Moreover, the NC group showed a significant reduction of caspase-3 levels in comparison to the NV group. In this case the effect was so strong that caspase-3 levels in HV group were similar to those in HC, with no additive effect shown by combining hypothermia and CBD.

The Lac/NAA ratio was reduced in the HV group in comparison to NV group (Figure 5C). Also, the effect of hypothermia alone (HV group) was slightly greater than that of CBD alone (NC group) after HI. Administering the two treatments together resulted in an additive effect, the Lac/NAA ratio in the HC group being lower than that in the NC and HV groups.

## Discussion

Although not completely conclusive because of the short-term follow-up, this study suggests that CBD and hypothermia could complement each other in their protective mechanisms resulting in a reduced brain injury in hypoxic-ischemic newborn piglets. In fact, the combination of the two therapies leads to better results in terms of anti-excitotoxicity, anti-inflammation and anti-oxidation than those observed with either CBD or hypothermia alone.

Excitotoxicity, inflammation and oxidative stress, the “deadly triad” leading to HI-induced brain damage, are particularly damaging for the immature brain (Johnston et al., 2011; Drury et al., 2014; Juul and Ferriero, 2014). In particular, the high concentration and activity of glutamate receptors aggravate the deleterious effects of this excitatory amino acid in the immature brain (Johnston et al., 2011; Drury et al., 2014; Juul and Ferriero, 2014). Among various approaches to quantify the increase in glutamate content in the immature brain after a hypoxic-ischemic insult, the Glu/NAA ratio has been observed to be closely related to excitotoxic brain damage (Groenendaal et al., 2001). The relative paucity of antioxidants and excess of pro-oxidant substances (in particular, iron) determine the particular susceptibility of the immature brain to oxidative stress (Johnston et al., 2011). HI-induced increases in oxidative stress can be assessed by quantifying protein carbonylation, an irreversible type of oxidative damage leading to functional loss observed in brain cells after ischemia/reperfusion (Oikawa et al., 2009) and detectable very shortly after HI in the piglet brain (Mueller-Burke et al., 2008; Pazos et al., 2013). There is increasing evidence supporting the importance of inflammation in the pathophysiology of acute brain damage and its long-lasting impact on the immature brain after HI insults (Allan and Rothwell, 2001; Johnston et al., 2011; Drury et al., 2014; Juul and Ferriero, 2014). In particular, the increase in TNFα production after HI, which occurs as early as 1 h after insult, correlates with the extent of injured tissue and with clinical outcomes (Allan and Rothwell, 2001).

In this study, for the first time, we have presented evidence of hypothermia acting simultaneously on these three factors in HI-injured piglets. There are few studies directly demonstrating the anti-excitotoxic effect of hypothermia in HI-injured piglets. Using microdialysis studies, it was previously shown that hypothermia reduces the glutamate increase seen after HI (Thoresen et al., 1997). Here, we have reported that hypothermia attenuates the HI-induced increase in the Glu/NAA ratio. As for oxidative stress, it has been reported that hypothermia reduces the HI-induced increase in protein carbonyl formation seen in the piglet brain 6 h after HI (Mueller-Burke et al., 2008). This effect was confirmed in our experiments. In the present study we have found that hypothermia reduces TNFα production in the HI-injured piglet brain. There are no previous reports on the effects of hypothermia on TNFα production in the brain of newborn pigs *in vivo*. Other authors have reported that hypothermia reduces the LPS-induced increase in TNFα production seen in cultured microglial cells (Schmitt et al., 2007). It was found that gaseous hypothermia can modulate inflammation and phagocytosis in aged rat brain after cerebral ischemia (Joseph et al., 2012; Sandu et al., 2016). In addition, it is known that hypothermia modulates the serum cytokine increase observed in HI infants (Jenkins et al., 2012).

This work confirms that CBD is also a pleiotropic substance that modulates excitotoxicity (blocking the increase in Glu/NAA ratio), oxidative stress (preventing the increase in carbonylated proteins) and inflammation (reducing the increase in TNFα production) in HI-injured animals at short-term outcome. CBD reduces both the *in vitro* and *in vivo* release of glutamate, as previously demonstrated in slices of newborn mice forebrain exposed to oxygen-glucose deprivation (Castillo et al., 2010), and in the brain of HI rats (Pazos et al., 2012). This compound is a potential antioxidant either due to its molecular properties (Hampson et al., 1998) or because it can modulate the expression of iNOS and free radicals after HI (Johnston et al., 2011; Juul and Ferriero, 2014). In previous studies, we have shown that the significant release of free radicals associated with iNOS after HI, is especially harmful in newborn mice (Castillo et al., 2010). Further, it reduces the release of cytokines (IL-1, IL-6, TNFα) after an HI insult, as demonstrated in newborn rodents *in vitro* (Castillo et al., 2010) and *in vivo* (Pazos et al., 2012).

Interestingly, the combination of hypothermia and CBD produced an evident accumulative effect acting on the same characteristics of excitotoxicity, oxidative stress and inflammation compared to either CBD or hypothermia alone. This finding suggests that the mechanism by which CBD modulates those factors does not interfere with that of hypothermia. On the contrary, it suggests that CBD and hypothermia probably act complementarily on brain-damaging pathways during the same phase or at different time intervals. This indicates that CBD is potentially a good partner for hypothermia treatment. In addition, CBD showed a very similar profile to hypothermia regarding its effects on the parameters characterizing brain damage after HI. This indicates that CBD might be, in addition to an important adjunct to hypothermia, a valuable alternative whenever the latter is not feasible.

Previously, we have reported that HI injury induced an increase in the number of necrotic neurons in the cortex. Both hypothermia (Mueller-Burke et al., 2008) and CBD alone (Alvarez et al., 2008; Pazos et al., 2013) reduced the neuronal death in hypoxic-ischemic piglets, as early as 6 h after the injury. Caspases, including caspase-3, are good markers of the activation of apoptotic pathways (Drury et al., 2014). It has been demonstrated that CBD reduces the efflux of caspase-9 as observed in newborn mice forebrain slices exposed to oxygen glucose deprivation (Castillo et al., 2010), and hypothermia reduces caspase-3 expression in newborn rats and fetal sheep (Drury et al., 2014). However, hypothermia alone did not significantly reduce the number of TUNEL+ and caspase-3 immunoreactive cells in the cortex of piglets, 48 h after a hypoxic-ischemic insult (Faulkner et al., 2011). Although no additive effect on caspase-3 was observed, the disagreement between these and our results may be due to the different follow-up periods and/or the different methods used to induce HI brain damage.

Lactate accumulates in the brain after HI as a result of mitochondrial dysfunction, whereas NAA content decreases when the number of viable neurons and oligodendrocytes decreases; thus, the Lac/NAA ratio is an excellent indicator of hypoxic-ischemic brain injury (Vial et al., 2004; Munkeby et al., 2008). In hypoxic-ischemic piglets, Lac/NAA ratio is closely correlated with the results from TUNEL and microglial ramification studies (Faulkner et al., 2011). In our study, both CBD and hypothermia alone attenuated the HI-induced modification in Lac/NAA in the cortex, in agreement with previous reports (Faulkner et al., 2011; Pazos et al., 2013). Moreover, the combination of hypothermia and CBD produced the best protective effects, being the most effective treatment to reduce histological brain damage and the Lac/NAA ratio. Well-known neuroprotective agents, such as xenon (Chakkarapani et al., 2010; Faulkner et al., 2011) or melatonin (Robertson et al., 2013), have also been demonstrated to augment hypothermia neuroprotection in the brain of injured piglets. We cannot rule out the possibility that brain damage was somehow mitigated in NV animals, since the use of dopamine infusion could reduce brain damage in some experimental models of newborn pigs (Park et al., 2003). However, neither CBD nor the combination of the therapies were associated with acute hypotensive episodes that would require inotropic support. In addition, a remarkable recovery of aEEG amplitude was observed in HC but not in HV animals. A similar effect has been previously described for CBD in asphyxiated piglets (Alvarez et al., 2008; Pazos et al., 2013) in association with the improvement of brain metabolism and the reduction of brain edema and histological damage. In this case a possible additive effect of CBD and hypothermia could not be assessed since hypothermia led to the flattening of aEEG trace throughout the experiment, as previously reported in similar models (Robertson et al., 2013).

Although providing encouraging results, the main limitation of our work is the short duration of the follow-up after the HI insult. In immature brain, the initial HI insult is usually followed by a latent period lasting for 6–24 h. During this period the aforementioned “deadly triad” (excitotoxicity, inflammation and oxidative stress) initiates various pathological processes that eventually lead to a secondary deterioration due to delayed energy failure (Johnston et al., 2011; Drury et al., 2014; Juul and Ferriero, 2014). Once initiated, most of these processes are thought to be irreversible (Drury et al., 2014). Theoretically, therefore, neuroprotective strategies must be applied prior to the onset of such irreversible processes (Drury et al., 2014). That is why the window for initiating neuroprotective strategies, such as hypothermia, is up at the start of the latent period, which usually is 6 h following HI insult. Hence, studying whether a neuroprotective treatment can effectively modulate excitotoxicity, inflammation and oxidative stress before the latent phase is a necessary early step to establish whether such treatment can be considered potentially useful (Mueller-Burke et al., 2008). In this context, our data suggest that CBD combined with hypothermia has beneficial modulatory effects when applied at short term. However, since the aforementioned processes are still working beyond the latent phase spreading and aggravating the damage (Drury et al., 2014), neuroprotective strategies are administered for at least 48 h in experimental conditions (Chakkarapani et al., 2010; Faulkner et al., 2011; Robertson et al., 2013) or, as for hypothermia (Johnston et al., 2011; Drury et al., 2014; Juul and Ferriero, 2014), in the clinical practice. It has been demonstrated, for instance, that gaseous hypothermia has different therapeutic effects on aged rats after cerebral ischemia depending whether the treatment was administered for 24 or 48 h (Joseph et al., 2012; Sandu et al., 2016). Another limitation of our study is the short lapse between the end of the HI insult and the initiation of the treatment. This short lapse is useful in terms of studying the effects of the treatments on the different cellular processes initiated by HI but is unrealistic in clinical terms. Therefore, before considering this treatment for clinical use it would be necessary to determine whether the neuroprotective effects of cannabidiol combined with hypothermia still act throughout the secondary deterioration period, which will require further experiments extending the monitoring period beyond 48 h after HI and with a more prolonged lapse between HI and treatments. Finally, other interventions, like anesthesia, can also significantly modulate the damaging processes and interact in exactly the same way in all anesthetized animals acting as potential neuroprotective agents. However, ethical aspects preclude the elimination of the anesthesia/analgesia and their effects cannot be specified.

In conclusion, CBD and hypothermia applied at short term act on the same processes related to HI brain damage, modulating excitotoxicity, inflammation, and oxidative stress. These two therapies in combination do not compete which each other in modulating these processes, but rather produce additive effects, resulting in greater overall benefit. Whether or not this combination produces effective long-term additive neuroprotection should be tested using longer follow-up studies and a “realistic” delay of treatment.

## Author contributions

JM and FA have designed the experimental design in accordance with international ARRIVE guidelines. HL, MP, NM, MS, MA, JM, and FA have performed the *in vivo* experimental phase. HL, MP, AA, and MA have performed the *in vitro* experimental phase. AA, JM, and FA have contributed with reagents/materials/analysis tools. HL, JM, and FA have been responsible for data analysis. HL, MP, AA, NM, MS, MA, JM, and FA have collaborated and approved the final manuscript version.

## Funding

This research has been supported from Eusko Jaurlaritza and ISCIII-General SubDirectorate for Research Assessment and Promotion and the European Regional Development Funds (FEDER) “A way to build Europe.” This work was partially supported by grants from the Spanish Health Research Foundation PI1200852, and PI1200192 from ISCIII-General SubDirectorate for Research Assessment and Promotion and the European Regional Development Funds, from the Eusko Jaurlaritza IT773-13 and from GW Pharma Ltd. (GWCRI09119).

### Conflict of interest statement

JM has received a grants from GW Pharma Ltd. (GWCRI09119), but did not influence the study design, data collection, analysis, or interpretation, the writing of the report, or decisions regarding submission. The other authors declare that the research was conducted in the absence of any commercial or financial relationships that could be construed as a potential conflict of interest.
